# Interactions between homeostatic plasticity and statistical learning: A role for inhibition

**DOI:** 10.1016/j.conb.2025.103065

**Published:** 2025-06-20

**Authors:** Elisa Galliano, Tara Keck

**Affiliations:** 1 Department of Physiology, Development and Neuroscience, University of Cambridge, UK; 2 Department of Neuroscience, Physiology and Pharmacology, University College London, London WC1E 6DE, UK

## Abstract

Statistical learning, sensory-driven unsupervised learning of repeating patterns, must coexist with ongoing homeostatic plasticity that is responsible for the necessary balance of activity in the brain; however, the mechanisms that facilitate these interactions are not clear. While models of both statistical learning, a form of associative plasticity, and homeostatic plasticity have primarily focused on excitatory cells and their synaptic changes, inhibition may play a key role in facilitating the balance between homeostatic plasticity and statistical learning. Here, we review the inhibitory synaptic, cellular, and network mechanisms underlying homeostatic and associative plasticity in rodents and propose a model in which localized inhibition, provided by diverse interneuron types, supports both statistical learning and homeostatic plasticity, as well as the interactions between them.

Homeostatic plasticity ensures neural activity remains within a functional range over extended periods of time, a requirement of all circuits which is perhaps even more critical in sensory areas, where environmental stimuli constantly change across large orders of magnitude [1]. Without homeostatic compensatory mechanisms, systems become inherently unstable, resulting in either runaway activity or a quiescent circuit [2]. These essential homeostatic mechanisms must also act in conjunction with forms of associative and Hebbian plasticity that underlie learning, memory formation, and functional reorganization after changes in the sensory periphery. The combination of these plasticity mechanisms allows for flexibility in the circuit to encode novel experiences (associative plasticity) while avoiding extreme activity levels (homeostatic plasticity). One important form of associative learning is statistical learning−sensory-driven, unsupervised learning of statistical patterns of sensory inputs−which, by definition, relies on repeated presentations of the same stimuli. Consequently, it is likely slower than reinforcement or one-shot learning, suggesting there may be a convergence of timescales between statistical learning and the learning-induced homeostatic plasticity that it must coexist with. However, how statistical learning and homeostatic plasticity integrate to enable changes to the system while maintaining activity balance without hindering one another is not fully clear.

Experiments and models of both statistical learning and homeostatic plasticity in adult rodents have traditionally emphasized changes at excitatory synapses, particularly through mechanisms like synaptic scaling, and Hebbian long-term potentiation (LTP) and long-term depression (LTD) [1,3,4]. However, methodological advances in genetically targeting specific inhibitory interneuron subtypes for activity manipulation and plasticity read-outs have enabled novel experiments revealing their role in these forms of plasticity. Focusing on the rodent brain, here we review inhibitory plasticity mechanisms that are thought to support homeostasis, as well as associative and statistical learning, and propose that differential inhibitory plasticity processes may help mediate their interactions.

## Inhibitory plasticity mechanisms: homeostasis

Plasticity of inhibitory interneurons and inhibitory synapses has been shown to play a crucial, yet often underemphasized, role in homeostatic plasticity [5–7]. Traditionally induced by transient loss or over-representation of inputs (*e.g*. via surgical, chemical, or mechanical manipulation of the peripheral sensor; environmental enrichment; or the pharmacological or chemogenetic manipulation of local circuits) and studied as part of maintaining the critical balance between excitation and inhibition at the subcellular, cellular, and network levels [5,8], inhibitory plasticity can occur in a wide variety of ways.

At the level of synapses, the strength and number of inhibitory synapses onto excitatory neurons can undergo homeostatic changes in response to changes in sensory input via synaptic scaling-like mechanisms or Hebbian mechanisms such as shifting the LTP/LTD threshold for plasticity induction [4,9–12]. Either of these mechanisms could result in altered levels of inhibition onto these principal cells. Additionally, the excitatory and inhibitory synapses onto inhibitory neurons themselves can change as a result of changes to sensory input [13–15], which could also alter inhibitory cell activity due to increases or decreases in synaptic drive. Network-level activity is influenced by these changes in activity levels of individual inhibitory neurons or in the number of inhibitory neurons in olfactory and hippocampal circuits via adult neurogenesis [14,16–18]. Previous studies have shown that inhibitory neurons regulate small, local cortical networks through inhibition stabilization, where strong reciprocal excitatory−inhibitory connections help maintain balanced activity levels and provide a fast-acting form of homeostatic regulation [19–21].

A nonsynaptic mechanism that may underlie changes in activity levels in inhibitory neurons themselves is the modulation of intrinsic excitability, which is well suited to rapidly change activity levels [22]. Changes in excitability in inhibitory neurons has been shown to occur as a homeostatic response to decreased input in somatosensory [23], auditory [24], visual [25], and olfactory [26] cortical and subcortical areas. Because of its speed of implementation, sensitivity to changes in inputs including neuromodulatory inputs [27,28], and effectiveness in adjusting the output of the entire neuron, plasticity of intrinsic excitability can be highly effective at controlling homeostatic inhibition and disinhibition in individual inhibitory neurons and therefore also in the network.

Moreover, there are a number of different inhibitory subtypes, including (but not limited to) parvalbumin (PV), somatostatin (SOM), vasoactive intestinal peptide (VIP)−, calretinin- (CR), and calbindin- (CB) positive neurons, as well as dual releasers of GABA and monoamines or neuromodulators [29,30]. Different inhibitory subtypes are known to target other inhibitory subtypes, which can facilitate disinhibitory activity, as well as different parts of the dendritic tree, the soma, or the axon initial segment (AIS) of principal cells [31–34]. Given that homeostatic regulation can occur at the AIS [26,35], within dendrites [13,36], at cells [37], and in small networks [19], activity changes in inhibitory subtypes targeting any of these spatial scales could facilitate homeostatic plasticity. Because of their different postsynaptic targets, activity changes in different subtypes would have drastically different effects on overall activity, depending on the nature of their connections.

## Inhibitory plasticity mechanisms: learning and associative plasticity

Inhibition is also proposed to play an important role in plasticity induction associated with learning. In both human [38] and animal models [39], inhibition has been proposed to gate learning, with a reduction in inhibition levels being associated with increased levels of learning and associative plasticity at excitatory synapses. Similar observations have been made for homeostatic plasticity occurring following the loss of peripheral input, with a reduction of inhibitory levels preceding excitatory synaptic plasticity proposed to be associated with fast compensation and functional remapping [6,40]. These permissive homeostatic changes in inhibition include reduced excitatory input onto inhibitory neurons [40,41], decreased turnover of adult-born inhibitory neurons [42], and reduced inhibitory inputs onto excitatory neurons [40,43,44], all of which lower inhibitory synaptic drive onto excitatory neurons. While historically there have been fewer studies mechanistically linking inhibitory plasticity with behavioral-level associative learning, more recent work has started to strongly link changes in plasticity in inhibitory cells with disinhibition of principal neurons and the overall network [45–48]. Supporting this idea, it has been widely demonstrated that following sensory changes and learning induction, inhibitory plasticity precedes excitatory plasticity, with inhibitory changes acting on a faster time course [25,49].

The consistency of these results across different plasticity induction paradigms suggests that this may be a general principle. Therefore, reducing inhibition, whether homeostatically or not, through changes to inhibitory synapses and neurons may also facilitate statistical learning, though this remains untested. Furthermore, different inhibitory subtypes have been shown to have unique plasticity profiles and play different roles in circuit computation and reinforcement learning [50–52]; however, the specificity of their plasticity in statistical learning remains unexplored.

## A potential role for inhibition in homeostasis and statistical learning

**Figure 1:**
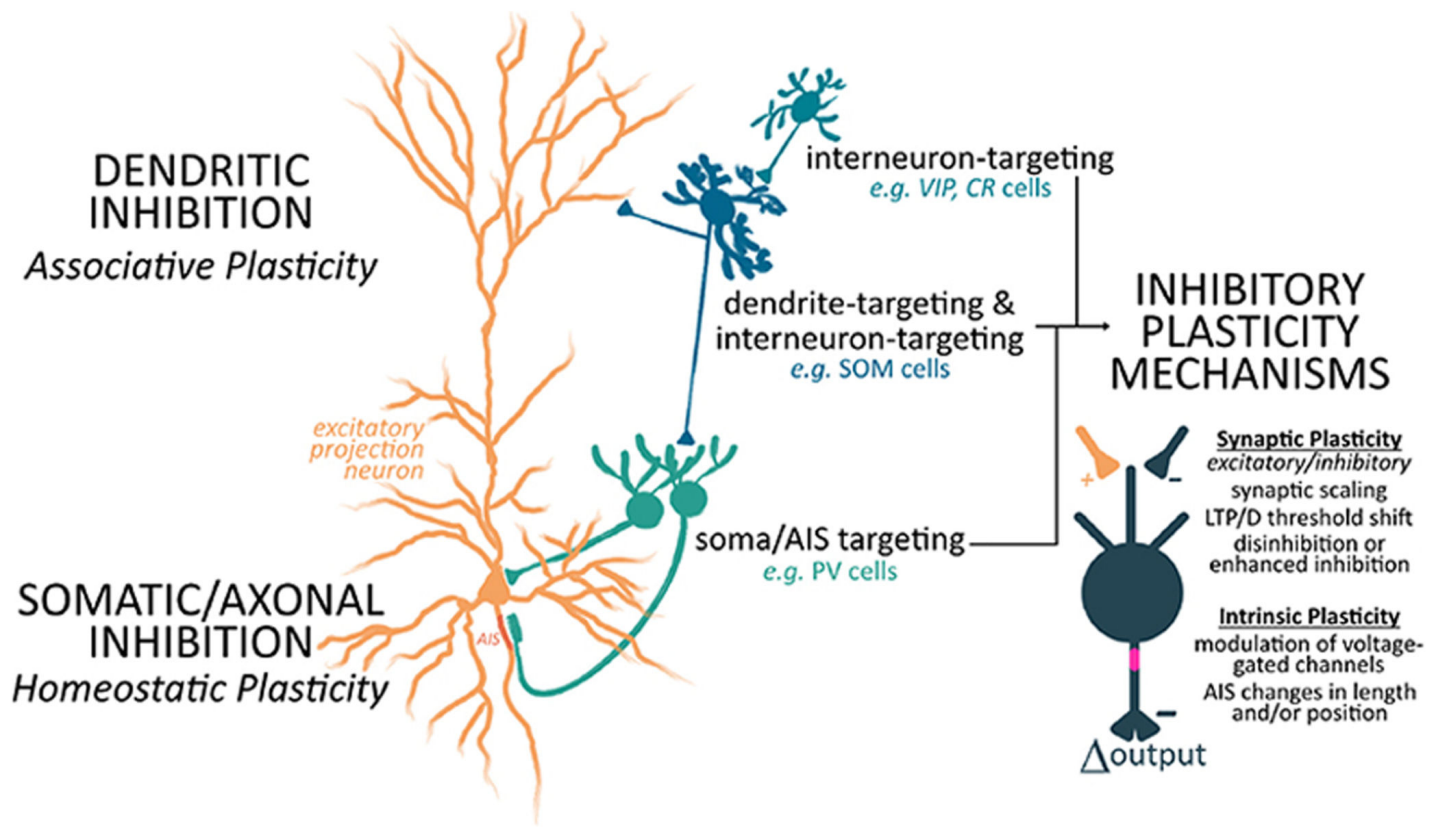
Schematic representation of the working hypothesis that localized inhibitory plasticity, mediated by diverse plasticity mechanisms, may enable the synergistic coexistence of associative plasticity and statistical learning with maintaining network homeostasis. The indicated examples of neuronal subtypes are found in the cortex and hippocampus, but a similar circuit design—albeit with different cell names and makers—applies broadly across the brain. AIS, axon initial segment; VIP, vasoactive intestinal peptide; CR, calretinin; SOM: somatostatin; PV, parvalbumin; LTP/LTD, long-term potentiation/long-term depression.

While inhibitory homeostatic plasticity is well studied at the molecular and cellular level, little is known about how inhibition mechanistically supports statistical learning. Thus, we venture to extrapolate from the existing literature and propose a deliberately speculative hypothesis on how statistical learning, inhibition, and homeostasis may intersect. One possibility is that different inhibitory subtypes mediate homeostatic plasticity and statistical learning respectively (Figure 1), which would support these two complementary forms of plasticity occurring in tandem, while minimizing conflict. For example in the cortex, cell- and network-level homeostatic balance could be regulated through changes to interneurons, such as PV cells, that typically target the excitatory cells’ soma [32]. PV cells have been shown to increase their selectivity to match that of excitatory pyramidal cells during visual reinforcement learning [50], with PV−pyramidal cell coupling potentially having a stabilizing effect on network activity. Similar changes in other brain areas could also entrain soma-targeting interneurons to strengthen coupling with excitatory cells during learning-related increases in activity, which could in theory help balance network activity via inhibitory−excitatory reciprocal interactions. Additionally, a subset of PV cells, *i.e*. corticohippocampal chandelier cells and cerebellar basket cells, are known to target the AIS of pyramidal/Purkinje cells, which affects the action potential threshold and as a result, cell and network activity [53]. Changing inhibition levels onto the AIS would provide another PV-specific way in which cellular and network activity can be homeostatically regulated [54]. Complementing PV plasticity, a reduction of inhibition from interneurons targeting the dendrites of excitatory neurons, for example, bulbar granule cells and cortical SOM cells [32,55], could be achieved either directly or indirectly through increased inhibitory drive from VIP cells onto SOM cells [56]. This reduction could gate the associative or Hebbian synaptic plasticity that is proposed to underlie statistical learning, similar to what has been observed for other forms of learning and plasticity [40,50].

We propose this spatial compartmentalization of regulation of excitatory cells by different inhibitory subtypes may facilitate complementary statistical learning and homeostatic plasticity, with dendrite-targeting inhibitory neurons gating excitatory plasticity in local compartments, as has been observed in learning and functional reorganization [39,40,43,44], and PV-like inhibitory neurons homeostatically regulating activity levels at the soma or AIS, affecting cellular and network activity [52,54]. While these two forms of plasticity are unlikely to be strictly restricted to these spatial compartments, this framework may generally provide a mechanism by which these forms of plasticity coexist. Furthermore, these homeostatic changes in inhibition are unlikely to be operating in isolation and likely occur in tandem with other homeostatic mechanisms in excitatory neurons, such as intrinsic excitability modulation, synaptic scaling, and shifting the threshold for LTP/LTD induction. These mechanisms have been discussed elsewhere and have been shown to globally regulate synaptic strength while allowing for localized associative or Hebbian plasticity that are thought to underlie learning [1,3].

While relatively few experimental studies have examined the role of inhibition in statistical learning, many associative learning and statistical learning models do contain inhibitory layers [57,58]. Furthermore, numerous models have demonstrated that inhibition stabilization is a key feature in the cortex, with recurrent networks of excitatory and inhibitory cells showing strong coupling that creates stable activity levels independent of the level of sensory stimulation [20,59,60]. To date, models of statistical learning or homeostasis have not typically incorporated spatially compartmentalized inhibitory inputs that would be associated with particular inhibitory subtypes. A model of statistical learning that includes localized inhibition, along with homeostatic compensatory components including direct modulation of intrinsic excitability, could be a critical first step for testing the role of inhibitory subtypes in statistical learning, homeostasis, and the interactions between the two.

## Data Availability

No data was used for the research described in the article.
